# Outcomes and Prognostic Factors of Intravitreal Bevacizumab Monotherapy in Zone I Stage 3+ and Aggressive Posterior Retinopathy of Prematurity

**DOI:** 10.1155/2015/102582

**Published:** 2015-09-27

**Authors:** Simona Delia Nicoară, Constanța Nascutzy, Cristina Cristian, Iulian Irimescu, Anne Claudia Ștefănuț, Gabriela Zaharie, Tudor Drugan

**Affiliations:** ^1^Department of Ophthalmology, “Iuliu Hațieganu” University of Medicine and Pharmacy, 8 Victor Babeș Street, 400012 Cluj-Napoca, Romania; ^2^“Alfred Rusescu” Institute for Mother and Child Care, 120 Lacul Tei Boulevard, 020395 Bucharest, Romania; ^3^Department of Neuroscience, “Iuliu Hațieganu” University of Medicine and Pharmacy, 8 Victor Babeș Street, 400012 Cluj-Napoca, Romania; ^4^Department of Ophthalmology, Emergency County Hospital, 3-5 Clinicilor Street, 400006 Cluj-Napoca, Romania; ^5^Department of Neonatology, “Iuliu Hațieganu” University of Medicine and Pharmacy, 8 Victor Babeș Street, 400012 Cluj-Napoca, Romania; ^6^Department of Medical Informatics and Biostatistics, “Iuliu Hațieganu” University of Medicine and Pharmacy, 8 Victor Babeș Street, 400012 Cluj-Napoca, Romania

## Abstract

*Purpose*. This study aims to evaluate the regression of retinopathy of prematurity (ROP) after one intravitreal injection of bevacizumab and the factors that influenced it. *Methods*. This retrospective case series was carried out at the “Iuliu Hațieganu” University of Medicine and Pharmacy, Cluj-Napoca, Romania. It includes all the consecutive infants treated for ROP with one intravitreal bevacizumab injection, from January 1, 2009, throughout July 31, 2013. The follow-up continued for 60 weeks after injection. We recorded ROP classification, regression, gender, gestational age, birth weight, postnatal age and postmenstrual age at treatment, and pregnancy type. Regression was analyzed according to each of the abovementioned factors, with the program IBM SPSS 20 (Chicago, Illinois, USA). *Results*. This study includes 74 eyes of 37 infants of which 52 had aggressive posterior ROP (70.27%) and 22 had zone I stage 3+ ROP (29.72%). One week after the bevacizumab injection, ROP regressed in 63 eyes (85.13%), with a statistically significant higher rate in zone I stage 3+ ROP (100%), as compared with aggressive posterior ROP (78.84%) (*P* = 0.03). We recorded no complications subsequent to the intravitreal injections of bevacizumab. We identified no late retinal detachment. *Conclusion*. ROP regression rate after one intravitreal bevacizumab injection was 85.13%. This trial is registered with trial registration number IRCT2014101618966N2.

## 1. Introduction

Bevacizumab (Avastin; Genentech Inc., San Francisco, California, USA) is a full anti-VEGF human monoclonal antibody approved in 2004 by the FDA for the treatment of metastatic colon cancer and used off label in the treatment of neovascular retinal diseases, such as retinopathy of prematurity (ROP) [1, 2]. The first reports on the use of bevacizumab for ROP were published in 2007 and they presented the experience in aggressive posterior ROP (AP-ROP) [3–5]. This severe form of ROP progresses rapidly to retinal detachment and often has an unfavorable outcome with standard laser treatment [6–10]. The reports on bevacizumab in AP-ROP included a small number of infants, but the finding that the outcomes were better in comparison with the ones of laser brought them into the spotlight.

The first prospective case series was published in 2008 and proved neovascular regression after bevacizumab injection, in 17 of the 18 eyes included in the study [11].

BEAT-ROP (Bevacizumab Eliminates the Angiogenic Threat of Retinopathy of Prematurity) was the first prospective, controlled, randomized trial that investigated the effect of one dose of bevacizumab (0.625 mg) for stage 3+ ROP, without combining laser and bevacizumab in the same infant. The recurrence rate in infants following bevacizumab was significantly lower as compared to the laser recurrence rate, 4% versus 22%. Benefits of bevacizumab over laser were statistically significant in zone I ROP. Another important observation of the BEAT-ROP study was that the injection of bevacizumab allows continued peripheral vascularization into avascular retina. However, the BEAT-ROP study has some unresolved issues: safety, long-term outcomes, and appropriate dosage [12].

In a previous study, we reported an AP-ROP regression rate of 54.16%, following laser treatment [10]. Disappointed by these outcomes and encouraged by the results mentioned above, we started to use bevacizumab injections to treat AP-ROP and zone I stage 3+ ROP.

We report our experience with intravitreal bevacizumab over a 55-month period for the treatment of ROP.

## 2. Materials, Subjects, and Methods

This is a retrospective case series.

*Main Outcome Measures*. ROP regression and the outcome associated with the intravitreal injection of bevacizumab.

### 2.1. Setting

The work was conducted in accordance with the Declaration of Helsinki (1964). All the intravitreal injections were carried out by the same ophthalmologist in the Departments of Neonatology and Ophthalmology belonging to the “Iuliu Hațieganu” University of Medicine and Pharmacy from Cluj-Napoca and in the Departments of Neonatology belonging to the “Saint Pantelimon” and “Polizu” Hospitals from Bucharest, Romania. The patients were enrolled after obtaining the informed consent from the parents/tutors. The study was approved by the Ethics Committee of the “Iuliu Hațieganu” University of Medicine and Pharmacy.

### 2.2. Study Sample

We analyzed retrospectively the files of all the consecutive infants with AP-ROP and zone I stage 3+ ROP who were treated by one intravitreal bevacizumab injection, from January 1, 2009, throughout July 31, 2013.

### 2.3. Screening Protocol

The treated premature infants came from a screening program that included the preterm newborns who met the following criteria: gestational age (GA) less than or equal to 33 weeks or birth weight (BW) less than or equal to 1500 grams. In the screening for ROP the premature infants with GA of more than 33 weeks and with BW of more than 1500 grams were also included, if other risk factors were associated: prolonged oxygen administration with saturation over 93%, repeated transfusions, sepsis, and necessity of more than 6 days of mechanical ventilation for the cardiorespiratory support. The first eye exam was performed at 4 weeks after birth in all the premature infants.

### 2.4. ROP Classification

We classified our cases according to the ICROP revisited [13]. This classification keeps the ICROP elaborated in 1984 [14], which takes into account the zone, the extension, the stage, and the presence/absence of “plus” disease and adds the definition of “pre-plus” disease and of the aggressive posterior ROP (AP-ROP). AP-ROP was defined as extreme vessel dilation and tortuosity in 4 quadrants, direct arteriovenous shunting, flat neovascularization, and rapid evolution, without following stage 1 to 3 progression [13].

### 2.5. Medical Intervention

A dose of bevacizumab of 0.625 mg in 0.025 mL was injected in each eye from the study sample, according to the following protocol: the pupils were dilated with a mixture of tropicamide 0.5% and phenylephrine 0.5%. Anesthesia was achieved with 0.5% proparacaine hydrochloride administered topically, 3 times, every 2 minutes before the injection. Every eye was prepared in a sterile manner using 5% povidone iodine which was also instilled in the eye 3 minutes prior to the injection. A nurse held the infant's head during the procedure and 0.025 mL of bevacizumab (0.625 mg) was injected in pars plicata, 1.5–1.75 mm away from the limbus, with a 30 G needle, perpendicularly on the globe initially and then slightly directed toward the center of the eyeball. After the injection, the patients received topical tobramycin, 5 times/day, for 3 days. The patients were reexamined the next day and then every week to monitor the regression of the disease. The follow-up continued for 60 weeks, every 2 weeks initially and then every 3 weeks, until full vascularization of the retina was observed. Full vascularization was defined as follows: vascularization as far as it would develop without an active component or clinically significant tractional elements. All the patients were followed by the pediatricians for long-term systemic complications. All the intravitreal injections were carried out by the same ophthalmologist and the examinations were performed by three ophthalmologists experienced in ROP.

We considered the following to be signs of regression: the pupil dilation, the disappearance or decrease of retinal vessel tortuosity and neovascularization, and the growth of the normal retinal vessels toward the peripheral retina. The treatment failure of ROP was defined according to the following criteria: the persistence/reappearance of plus disease and of retinal neovascularization and the progression toward retinal detachment. In all these latter situations, conventional laser photocoagulation of the retina was carried out.

Pictures of the retina before and after bevacizumab injections were taken with a Ret Cam (Clarity Medical System, Pleasanton, California, USA).

The following data were recorded: gender, GA, BW, zone and stage of ROP, other ocular findings (persistence of fetal vasculature, vitreous hemorrhage, and undilated pupil), postnatal age (PNA) and postmenstrual age (PMA) at treatment, type of pregnancy (unique, multiple), complications, and follow-up period.

### 2.6. Visual Outcome Assessment

Visual acuity (VA) testing was performed at 60 weeks by a trained pediatric ophthalmologist using Teller acuity cards (Stereo Optical Company, Inc., Chicago, IL) at a test distance of 40 cm. VA data were compared with available data of normal VA ranges for full-term infants. We considered as normal a VA within one or two octaves or greater than two octaves of the lower limit of the normal range. In cases with ROP progression towards retinal detachment, VA was categorized as unrecordable.

### 2.7. Statistical Analysis

We performed statistical analysis with the program IBM SPSS 20. Chi-square test was performed in order to verify the observed distributions in the contingency tables. If the theoretical distribution had less than 2 cases per cell we preferred the *P* value calculated with Fisher's exact test. The *P* value < 0.05 was considered statistically significant. Means comparisons were performed with Student's *t*-test because data were normally distributed and the number of cases was relatively low. Variance was tested with Levene's test.

## 3. Results

### 3.1. Demographic and Clinical Characteristics of the Study Sample

Our study was performed on 74 eyes with zone I stage 3+ ROP and AP-ROP belonging to 37 premature infants, treated by intravitreally injected bevacizumab (Table 1). The demographic and clinical characteristics of the premature infants are presented in Table 1.

All of the infants had a history of supplemental oxygen and mechanical ventilation use. None of the infants had a history of laser prior to the bevacizumab injection. Within the study sample, 17 infants were males (45.94%), 20 were females (54.05%), 29 were coming from unique pregnancies (78.37%), and 8 were coming from multiple pregnancies (21.62%). The mean GA was 28.89 ± 1.88 weeks (range 26–34 weeks) and the mean BW was 1218.35 ± 326.33 grams (range 800–2300 grams) (Table 1).

The ROP classification within the study sample is presented in Table 2.

Of the 37 prematures, 26 had bilateral aggressive posterior disease (AP-ROP) (70.27%) and 11 had bilateral zone I stage 3+ ROP (29.72%). We noted the persistence of fetal vasculature in 10 eyes (13.51%), undilated pupil in 7 eyes (9.45%), and vitreous hemorrhage in 4 eyes (5.71%), all within the AP-ROP group.

The most important data of each preterm newborn included in this study are illustrated in Table 3.

### 3.2. Outcomes of ROP after Intravitreal Bevacizumab Injections

One week after the bevacizumab injection, ROP regressed in 63 eyes (85.13%) (Table 1). ROP regressed bilaterally in 30 cases (81.08%), regressed unilaterally in 3 cases (8.10%), and progressed bilaterally in 4 cases (10.81%).

#### 3.2.1. Structural Outcomes at 60 Weeks

In all of the 11 eyes that failed to regress after one intravitreal injection of bevacizumab, laser photocoagulation was carried out at 7–10 days after the intravitreal bevacizumab, with good evolution in 9 of the 11 eyes. At 60 weeks, retina was attached completely in 72 eyes (97.29%) and totally detached (stage V) in 2 eyes (2.70%). None of the cases in this series progressed towards bilateral retinal detachment.

#### 3.2.2. Functional Outcomes at 60 Weeks

From the 72 eyes with good structural outcomes at 60 weeks, 68 had VA within normal limits (94.44%). All the 4 eyes with lower than normal VA come from the AP-ROP subgroup with laser addition.

### 3.3. Individual Factors Associated with the Outcomes after Bevacizumab Injections

We analyzed the following individual factors in correlation with the evolution after the intravitreal injection of bevacizumab: ROP type, gender, type of pregnancy, GA, and BW. Bevacizumab administered as one intravitreal injection was followed by ROP regression in all the 22 eyes with zone I stage 3+ ROP (100%) and in 41 of the 52 eyes with AP-ROP (78.84%) (Table 3). Fischer's exact test proved that the success rate was significantly higher in stage 3 zone I ROP, as compared to AP-ROP (*P* = 0.03).

Within the group of girls (40 eyes), ROP regressed in 33 eyes (82.50%) and within the group of boys (34 eyes), ROP regressed in 30 eyes (88.23%). The difference between genders was not statistically significant (*P* > 0.05).

Of the 37 pregnancies, 29 were unique and 8 were multiple. Within the unique pregnancies group (58 eyes), ROP regressed in 47 eyes (81.03%) and within the multiple pregnancies group (18 eyes), ROP regressed in all the 16 eyes (100.00%). The difference was not statistically significant (*P* > 0.05).

An independent *t*-test was conducted to determine if there was a difference between the mean GA of the premature infants with favorable or unfavorable outcome after bevacizumab injections. The results of Levene's test *F*(72) = 0.43, *P* = 0.51 indicate that the variances of the two populations (with favorable and unfavorable outcome) were approximately equal. Thus, the *t*-tests assuming equal variations were used. There was no statistically significant difference between the mean GA of the premature infants with favorable (*n* = 63; *M* = 28.94; SD = 1.86) and unfavorable (*n* = 11; *M* = 28.64; SD = 2.06) outcome, *t*(72) = 0.48, *P* = 0.62. The 95 confidence interval was −0.934 to 1.534.

An independent *t*-test was conducted to determine if there was a difference between the mean BW of the premature infants with favorable or unfavorable outcome after bevacizumab injections. The results of Levene's test *F*(72) = 0.66, *P* = 0.41 indicate that the variances of the two populations (with favorable and unfavorable outcome) were approximately equal. Thus, the *t*-tests assuming equal variations were used. There was no statistically significant difference between the mean BW of the infants with favorable (*n* = 63; *M* = 1219.52 g; SD = 341.75 g) and unfavorable (*n* = 11; *M* = 1211.64; SD = 230.65) outcome after bevacizumab therapy, *t*(72) = 0.73, *P* = 0.94. The 95 confidence interval was −206.153 to 221.928.

### 3.4. Timing of Bevacizumab Injections

We evaluated the moment of bevacizumab injections according to two parameters: PNA and PMA at treatment. PNA at the moment of bevacizumab injection varied on our series between 4 and 10 weeks (mean 6.22 ± 1 weeks) (Table 1). An independent *t*-test was conducted in order to determine if the mean PNA at treatment was significantly different between the favorable and unfavorable outcome subgroups. The results of Levene's test *F*(72) = 0.05, *P* = 0.82 indicate that the variances of the two populations (with favorable and unfavorable outcome) were equal. Thus, the *t*-tests assuming equal variations were performed. The PNA at treatment did not differ significantly between the favorable (*n* = 63; *M* = 6.11 w; SD = 1.38 w) and unfavorable (*n* = 11; *M* = 6.82 w, SD = 1.47 w) outcome subgroups, *t*(72) = −1.55, *P* = 0.12. The 95 confidence interval was −1.615 to 0.201. PMA at treatment varied between 31 and 38 weeks (mean 35.11 ± 2 weeks) (Table 2). An independent *t*-test was conducted in order to determine if the mean PMA at treatment was significantly different between the favorable and unfavorable outcome subgroups. The results of Levene's test *F*(72) = 0.02, *P* = 0.88 indicate that the variances of the two populations (with favorable and unfavorable outcome) were assumed to be approximately equal. Thus, the *t*-tests assuming equal variations were used. The PMA at treatment did not differ significantly between the favorable (*n* = 63; *M* = 35.05 w; SD = 1.59 w) and unfavorable (*n* = 11; *M* = 35.45 w; SD = 1.57 w) outcome subgroups, *t*(72) = −0.78, *P* = 0.43. The 95 confidence interval was −1.441 to 0.628.

**Figure 1:**
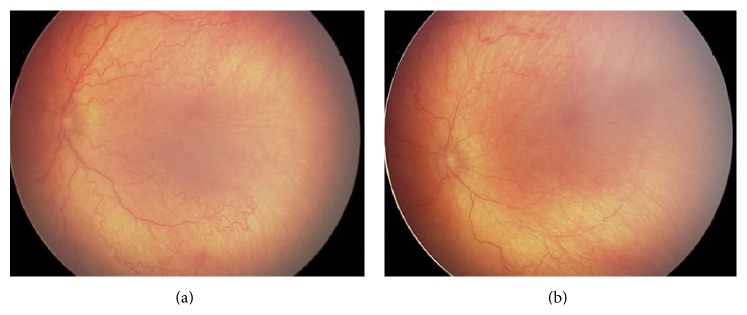
(a) AP-ROP prior to the bevacizumab injection: male infant, BW 1000 g, GA 28 w, treated with intravitreal bevacizumab at PMA of 34 weeks and PNA of 6 weeks. (b) Fundus photography 5 days after bevacizumab injection with venous dilatation and arteriolar tortuosity improvement in the posterior pole, showing plus disease regression.

Figures 1(a) and 1(b) illustrate a case of AP-ROP just prior to the bevacizumab injection (a) and 5 days after the injection (b).

The immediate good outcome of this case is revealed by the significant improvement of “plus” disease and the decrease of retinal vessel tortuosity.

**Figure 2:**
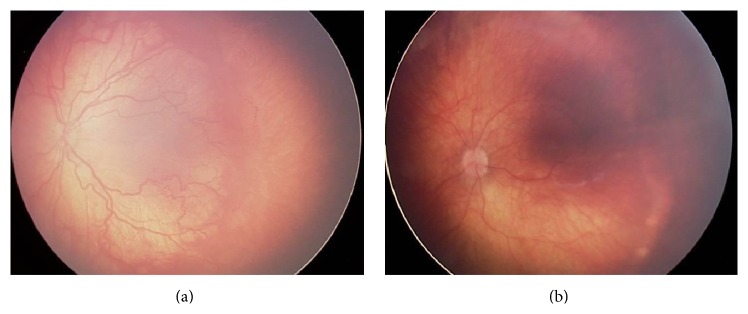
(a) Zone I disease prior to the bevacizumab injection: female infant, BW 2050 g, GA 28 w, treated with intravitreal bevacizumab at PMA of 34 weeks and PNA of 6 weeks. (b) The same case as in (a), 8 weeks after the intravitreal injection of bevacizumab showing ROP regression.

Figure 2 presents a case with zone I disease prior to the injection of bevacizumab (a) and 8 weeks after the injection (b). “Plus” disease disappeared, the ridge regressed, and the retinal vessels continued their normal growth towards the periphery.

We recorded no systemic complication subsequent to the intravitreal administration of bevacizumab in our series. No cataracts, endophthalmitis, or retinal detachments produced by the intraocular injections were identified in our series. The final anatomic outcome (after having added the laser photocoagulation of the retina) proved the bilateral attached retina in 35 cases (94.59%) and the unilateral attached retina in 2 cases (5.40%). No case ended up with bilateral retinal detachment.

## 4. Discussion

### 4.1. Rationale for Anti-VEGF Treatment in ROP

The basis for the intravitreal administration of anti-VEGF agents as treatment for ROP is the evidence that the concentration of VEGF is increased in the vitreous of infants with ROP [15, 16]. Bevacizumab blocks the molecules of VEGF already in the vitreous and it also inhibits the production of new ones. This explains the better and more rapid outcomes as compared to laser [1]. It was shown that ROP ceases within 48 h after the intravitreal administration of bevacizumab [17]. We observed the regression of “plus” disease even at 24 hours after the injection in our series. Bevacizumab is less destructive and more oriented towards the pathogenesis of ROP as compared to laser [18]. Unlike with laser, the retinal vascularization continues after the intravitreal injection of bevacizumab. We followed all the patients for 60 weeks and we observed the development of retinal vessels up to the periphery in all our cases with good outcomes after bevacizumab intravitreal injections. Moreover, according to the BEAT-ROP study, the intravitreal administration of bevacizumab is not associated with cystoid macular edema [12]. This finding is confirmed by our series.

As opposed to the laser treatment, the intravitreal injection of bevacizumab is shorter, easier, accessible, and less expensive; it can be performed if the pupils are small and/or vitreous hemorrhage is associated and it is more efficient than laser in zone I ROP. It was not our goal to compare the results of bevacizumab with laser. However, we found that the primary intravitreal injection of 0.025 mL (0.625 mg) of bevacizumab was followed by the regression of ROP in 78.84% of the AP-ROP eyes. In the lasered AP-ROP cases, we found regression in 53.84% [10].

### 4.2. Indications of Intravitreal Bevacizumab in ROP according to Our Experience

In our series, we used bevacizumab intravitreally in AP-ROP and in zone I stage 3+ ROP. In AP-ROP, our choice was motivated by our poor outcomes following laser treatment. As mentioned above, in our previous study, we registered regression rates of AP-ROP after laser treatment of 53.84% [10].

In zone I stage 3+ ROP, we used bevacizumab instead of laser, despite the good results of the laser treatment that we previously reported [10]. Our choice was motivated by the much longer duration of the laser treatment and its association with significant loss of visual field in this posterior location of the disease.

### 4.3. Demographic and Clinical Characteristics of the Study Sample

ROP is rare in infants with BW > 2000 g. On our series, we identified 2 infants with severe ROP, with BW over 2000 g. Also, 6 infants had BW over 1500 g, outside the screening criteria for ROP that are used in the USA. Nine of the 37 prematures had GA higher than 30 weeks, which is the limit for screening in the USA. This observation proves that, in each region, specific criteria for ROP screening must be applied. Older and bigger babies in developing and middle-income countries are known to develop significant ROP and possibly oxygen-induced retinopathy [19]. Larger and more mature babies developed severe forms of ROP disease in our series compared to screening criteria used in other countries (USA, Canada, and UK). This may suggest that possibly other predisposing factors not described in this study play a role in this aggressive presentation of ROP disease.

### 4.4. Timing of Intravitreal Bevacizumab Injections for ROP

The exact therapeutic window during which intravitreal bevacizumab is effective is not precisely known. The most important decisional factors are the PMA at treatment and the degree of membrane formation [18]. The PMA helps us evaluate the likely proportion between the angiogenic and fibrotic growth factors [18]. If intravitreal bevacizumab is administered too late, the contraction of membranes with subsequent acceleration of retinal detachment can result [20]. In all the premature infants included in this series, the initial eye exam had been done at 4 weeks after birth. None of the severe cases can be attributed to the failure in early diagnosis. In our series, the mean PMA at treatment was 35.11 weeks, with no statistically significant difference between the favorable (35.05 weeks) and unfavorable (35.45 weeks) outcome groups.

In the neovascular retinal diseases of the adult (age-related macular degeneration and diabetic retinopathy), there is a continuous release of VEGF [1]. As opposed to this situation, in ROP there is a single burst of VEGF that initiates the retinal neovascularization. Therefore, the repeat of injection appears unnecessary in ROP [1]. In all the cases included in this series, we performed one single bevacizumab injection in each eye.

### 4.5. Outcomes

The only factor that influenced the outcomes of bevacizumab monotherapy in our series was the ROP type. ROP regressed in all the eyes with zone I stage 3+ ROP (100%) and in 78.84% of the eyes with AP-ROP after one intravitreal bevacizumab injection (*P* = 0.03). The outcomes in our series were not influenced by gender, type of pregnancy (unique or multiple), GA, BW, and PNA and PMA at treatment.

Recent studies proved that VEGF is not the only growth factor upregulated in the eye and therefore its inhibition may not induce the ROP regression in all cases. Other growth factors involved in ROP pathogeny are insulin-like growth factor, angiopoietin-1, and angiopoietin-2 [2, 16]. These data could partially explain the failures in our series.

Within the group of eyes with good structural outcomes (72 eyes), 94.44% had normal VA at 60 weeks. This observation allows us to affirm that if the structural result was successful, the potential for normal VA development was high in our series.

### 4.6. Safety

All the patients in the study sample were followed by pediatricians for systemic side effects related to bevacizumab, regarding the development of the brain, lungs, kidneys, and skeleton, and no abnormalities were reported. No local side effects, such as cataract, endophthalmitis, vitreous hemorrhage, and retinal detachment related to the intraocular injection, were identified in our series. ROP progression in 11 AP-ROP eyes was probably not due to the bevacizumab injection but to its inefficacy.

Still, there are concerns regarding the long-term effects of bevacizumab, the probability of the substance to leave the eye and the delayed-onset retinal detachment [15, 21].

In the eye, VEGF is not only an angiogenic factor, but also a neural survival one. Therefore, the suppression of the normal development of neural retinal components by the anti-VEGF therapy is suspected [15]. Histopathological studies, performed both on a rabbit model and in a very premature infant, proved that bevacizumab had no adverse effect on the growing and development of the eye [22, 23].

In the eyes with ROP, there is the breakdown of the blood-retinal barrier. As a consequence, the anti-VEGF substances might leave the vitreous and get into the systemic circulation, reducing the serum VEGF level [15]. Laser photocoagulation of the retina destroys the natural retinal barrier, enhancing the exit of bevacizumab from the choroidal vessels into the blood. Subsequently, these infants are at a higher risk of systemic effects [1]. This is the reason why we used bevacizumab as the first-choice treatment, not subsequently to the laser therapy. At the moment when anti-VEGF therapy is administered, the infant is still in the process of organogenesis and the VEGF is necessary for the development of the brain, lungs, kidneys, and skeleton. Therefore, the injection of the minimal effective dose and the use of an anti-VEGF agent with the most rapid systemic clearance are recommended [15]. A 2007 report on the pharmacokinetics of bevacizumab found that the half-life of 1.25 mg is 4.32 days in a rabbit eye. In addition, small amounts were detected in the serum and the fellow eye. However, humans have a larger serum compartment than rabbits and, therefore, systemic exposure may be less [24]. In adults, the intrinsic serum elimination half-life of bevacizumab is 20 days. In children this is not known, but it is probably longer [25]. When we compare the dosage of bevacizumab which is used for the premature infants with the one in adults, we find that the concentration for bodyweight is very large in infants. The appropriate dosage that should be used is not well known [21].

Even if there has been no report on the possible systemic side effects of intravitreally administered bevacizumab, we do not know for sure the long-term side effects on a developing child. Larger and longer studies are needed, in order to fully address the systemic toxicity issues.

Bevacizumab changes the natural evolution of ROP; despite the initial resolution there are chances of recurrence that we must be aware of. The delayed-onset retinal detachment, 4–4.5 months after the injection, is explained by the incomplete regression of the fibrovascular proliferation that develops slowly into a tractional complex [26]. The practical conclusion of this observation is the necessity to extend the follow-up period. We followed all the infants in this series for 60 weeks after the intravitreal injection of bevacizumab. We report no late retinal detachment.

A recent study documented significant vascular and macular abnormalities at the age of 9 months, in the eyes that received intravitreal bevacizumab for ROP [27]. These findings were not identified in the laser treated eyes. Long-lasting implications of these abnormalities for visual function of the child need further investigation [27].

The limitations of our study are its retrospective nature and the lack of a concomitant control group. On the ground of our results, currently, bevacizumab is our treatment of choice in AP-ROP and zone I disease. We follow each patient for 15 months after the injection, in order to identify late onset retinal detachment. We still have questions to be answered, regarding the optimal dosing, timing, avoidance of unnecessary treatment, long-term effects, and safety.

## 5. Conclusion

ROP regression rate after one intravitreal bevacizumab injection was 85.13%. Our study proves that 100% of patients with zone I stage 3+ ROP and 78.84% of patients with AP-ROP regressed with a single injection of bevacizumab, without ocular or systemic complications. Eyes with zone I stage 3+ ROP were more likely to have regression of ROP with a single injection of bevacizumab than those with AP-ROP.

## Figures and Tables

**Table 1:** Demographic and clinical characteristics of the premature infants who received intravitreal bevacizumab as primary therapy for ROP.

Eyes (patients)	74 (37)
Male/female	17/20
Unique/multiple pregnancy	29/8
Mean gestational age (wk)	28.89
Mean birth weight (g)	1218.35
Mean injection time (postmenstrual age/postnatal age, wk)	35.11/6.22
Number of eyes (%) with regression after bevacizumab injection alone	63 (85.13)

**Table 2:** Classification of ROP in the eyes treated with intravitreal injections of bevacizumab.

ROP classification	Eyes (number)	Eyes (%)
AP-ROP^∗^	52	70.27
Stage 3 zone I with “plus”	22	29.72

^∗^AP-ROP = aggressive posterior ROP.

**Table 3:** Data of each premature infant treated by intravitreal injections of bevacizumab.

Case	Gender	GA (w)	BW (g)	ROP (OD/OS)	PNA (w)	PMA (w)	Outcome (OD/OS)
1	M	28	900	AP^∗^/AP	8	36	Good/bad
2	F	28	1000	AP/AP	5	33	Good/good
3	F	32	1800	AP/AP	4	36	Good/good
4	M	34	2300	3 I^∗∗^/3 I	4	38	Good/good
5	F	27	995	AP/AP	7	34	Good/good
6	F	30	1400	3 I/3 I	4	34	Good/good
7	M	30	1500	3 I/3 I	4	34	Good/good
8	F	26	1100	AP/AP	5	31	Good/good
9	F	28	990	AP/AP	6	34	Good/good
10	M	30	1650	3 I/3 I	7	37	Good/good
11	F	28	2050	3 I/3 I	6	34	Good/good
12	F	27	1100	AP/AP	8	35	Good/good
13	F	29	1140	AP/AP	8	37	Good/bad
14	M	30	1200	3 I/3 I	7	37	Good/good
15	F	27	970	AP/AP	6	33	Good/good
16	F	29	1070	AP/AP	6	35	Good/good
17	F	28	990	AP/AP	7	35	Good/good
18	M	28	1000	AP/AP	6	34	Good/good
19	F	31	1200	3 I/3 I	5	36	Good/good
20	F	31	1100	3 I/3 I	5	36	Good/good
21	F	30	1030	AP/AP	6	36	Good/good
22	M	28	990	AP/AP	7	35	Good/good
23	M	28	1100	AP/AP	7	35	Good/good
24	M	28	985	AP/AP	7	35	Good/good
25	M	31	1400	AP/AP	4	35	Bad/bad
26	M	32	1500	3 I/3 I	5	37	Good/good
27	M	31	1240	AP/AP	5	36	Good/good
28	F	31	1499	AP/AP	7	38	Bad/bad
29	M	27	1010	AP/AP	7	34	Good/good
30	F	26	1100	AP/AP	8	34	Bad/bad
31	F	27	950	AP/AP	7	34	Bad/bad
32	M	27	980	AP/AP	5	32	Good/good
33	M	28	1550	3 I/3 I	8	36	Good/good
34	F	27	800	AP/AP	10	37	Good/good
35	M	28	1390	AP/AP	7	35	Good/bad
36	F	31	1550	3 I/3 I	6	37	Good/good
37	M	28	1050	AP/AP	6	34	Good/good

^∗^AP = aggressive posterior; ^∗∗^3 I = stage 3 zone I.

## References

[1] Micieli J. A., Surkont M., Smith A. F. (2009). A systematic analysis of the off-label use of bevacizumab for severe retinopathy of prematurity. *American Journal of Ophthalmology*.

[2] Lin C., Chen S., Tseng C., Chang Y., Hwang J. (2012). Effects of ranibizumab on very low birth weight infants with stage 3 retinopathy of prematurity: a preliminary report. *Taiwan Journal of Ophthalmology*.

[3] Travassos A., Teixeira S., Ferreira P. (2007). Intravitreal bevacizumab in aggressive posterior retinopathy of prematurity. *Ophthalmic Surgery Lasers and Imaging*.

[4] Chung E. J., Kim J. H., Ahn H. S., Koh H. J. (2007). Combination of laser photocoagulation and intravitreal bevacizumab (Avastin) for aggressive zone I retinopathy of prematurity. *Graefe's Archive for Clinical and Experimental Ophthalmology*.

[5] Kusaka S., Shima C., Wada K. (2008). Efficacy of intravitreal injection of bevacizumab for severe retinopathy of prematurity: a pilot study. *British Journal of Ophthalmology*.

[6] O'Keefe M., Lanigan B., Long V. W. (2003). Outcome of zone 1 retinopathy of prematurity. *Acta Ophthalmologica Scandinavica*.

[7] Récsán Z., Vámos R., Salacz G. (2003). Laser treatment of zone I prethreshold and stage 3 threshold retinopathy of prematurity. *Journal of Pediatric Ophthalmology and Strabismus*.

[8] Kychenthal A., Dorta P., Katz X. (2006). Zone I retinopathy of prematurity: clinical characteristics and treatment outcomes. *Retina*.

[9] Early Treatment for ROP Cooperative Group (2003). Revised indications for the treatment of retinopathy of prematurity: results of the early treatment for retinopathy of prematurity randomized trial. *Archives of Ophthalmology*.

[10] Nicoară S.-D., Cristian C., Irimescu I., Stefanut A.-C., Zaharie G. (2014). Diode laser photocoagulation for retinopathy of prematurity: outcomes after 7 years of treatment. *Journal of Pediatric Ophthalmology and Strabismus*.

[11] Quiroz-Mercado H., Martinez-Castellanos M. A., Hernandez-Rojas M. L., Salazar-Teran N., Chan R. V. P. (2008). Antiangiogenic therapy with intravitreal bevacizumab for retinopathy of prematurity. *Retina*.

[12] Mintz-Hittner H. A., Kennedy K. A., Chuang A. Z. (2011). Efficacy of intravitreal bevacizumab for stage 3+ retinopathy of prematurity. *The New England Journal of Medicine*.

[13] International Committee for the Classification of Retinopathy of Prematurity (2005). The international classification of retinopathy of prematurity revisited. *Archives of Ophthalmology*.

[14] Garner A., Ben-Sira I., Deutman A. (1984). An international classification of retinopathy of prematurity. *British Journal of Ophthalmology*.

[15] Yang C.-H. (2012). Anti-vascular endothelium growth factor therapy for retinopathy of prematurity: a continuing debate. *Taiwan Journal of Ophthalmology*.

[16] Sato T., Shima C., Kusaka S. (2011). Vitreous levels of angiopoietin-1 and angiopoietin-2 in eyes with retinopathy of prematurity. *The American Journal of Ophthalmology*.

[17] Mintz-Hittner H. A., Kuffel R. R. (2008). Intravitreal injection of bevacizumab (avastin) for treatment of stage 3 retinopathy of prematurity in zone i or posterior zone II. *Retina*.

[18] Mintz-Hittner H. A. (2010). Avastin as monotherapy for retinopathy of prematurity. *Journal of AAPOS*.

[19] Gilbert C., Fielder A., Gordillo L. (2005). Characteristics of infants with severe retinopathy of prematurity in countries with low, moderate, and high levels of development: implications for screening programs. *Pediatrics*.

[20] Faia L. J., Trese M. T., Ryan S., Schachat A., Wilkinson C., Hinton D., Wiedemann P. (2013). Retinopathy of prematurity.

[21] Mataftsi A., Dimitrakos S. A., Adams G. G. W. (2011). Mediators involved in retinopathy of prematurity and emerging therapeutic targets. *Early Human Development*.

[22] Kong L., Mintz-Hittner H. A., Penland R. L., Kretzer F. L., Chévez-Barrios P. (2008). Intravitreous bevacizumab as anti–vascular endothelial growth factor therapy for retinopathy of prematurity: a morphologic study. *Archives of Ophthalmology*.

[23] Axer-Siegel R., Herscovici Z., Hasanreisoglu M., Kremer I., Benjamini Y., Snir M. (2009). Effect of intravitreal bevacizumab (Avastin) on the growing rabbit eye. *Current Eye Research*.

[24] Bakri S. J., Snyder M. R., Reid J. M., Pulido J. S., Singh R. J. (2007). Pharmacokinetics of intravitreal bevacizumab (Avastin). *Ophthalmology*.

[25] Avery R. L. (2012). Bevacizumab (Avastin) for retinopathy of prematurity: wrong dose, wrong drug, or both?. *Journal of AAPOS*.

[26] Jang S. Y., Choi K. S., Lee S. J. (2010). Delayed-onset retinal detachment after an intravitreal injection of ranibizumab for zone 1 plus retinopathy of prematurity. *Journal of AAPOS*.

[27] Lepore D., Quinn G. E., Molle F. (2014). Intravitreal bevacizumab versus laser treatment in type 1 retinopathy of prematurity. *Ophthalmology*.

